# Association of the triglyceride glucose index and its related indices with coronary artery disease risk: mediation by glycated haemoglobin

**DOI:** 10.3389/fendo.2026.1809007

**Published:** 2026-04-10

**Authors:** Yazhao Sun, Jie Huang, Xiao Yu, Jiejie Meng, Guijuan Qiu

**Affiliations:** 1 Department of Cardiology, Cangzhou People’s Hospital, Cangzhou, Hebei, China; 2 Department of Neurology Intervention, Cangzhou People’s Hospital, Cangzhou, Hebei, China; 3 Department of Emergency, Cangzhou People’s Hospital, Cangzhou, Hebei, China

**Keywords:** coronary artery disease, glycatedhemoglobin, mediation, obesity indices, triglyceride-glucose index

## Abstract

**Background:**

The Triglyceride-Glucose (TyG) index has been recognized as an independent predictor of cardiovascular disease (CVD) risk. However, the combined effect of TyG index with obesity indices, such as body mass index (BMI), waist circumference (WC), waist-to-height ratio (WHtR), conicity index (CI), weight-adjusted waist index (WWI), and body roundness index (BRI), on CVD risk remains inconsistent across different studies. This study aims to evaluate the relationship between TyG index and its related indices with the risk of coronary artery disease (CAD) and explore the mediating role of glycated hemoglobin (HbA1c) in this association.

**Methods:**

This study included patients who underwent coronary angiography for the first time. Logistic regression models, restricted cubic spline (RCS) regression, and threshold effect analysis were used to assess the relationship between the TyG index and its related indices with the risk of CAD. The area under the curve (AUC) for each index was calculated using receiver operating characteristic (ROC) curves, and AUC differences were compared using DeLong’s test. Stratified analysis was performed to validate the robustness of the results, and mediation analysis was conducted to evaluate the mediating role of HbA1c in the relationship between TyG index, its related indices, and CAD risk.

**Results:**

This retrospective observational study included a total of 3, 641 participants. After adjusting for covariates, the TyG index and its related indices were significantly positively associated with CAD (TyG: OR = 1.642, 95% CI = 1.447, 1.866; TyG-BMI: OR = 1.010, 95% CI = 1.007, 1.012; TyG-WC: OR = 1.392, 95% CI = 1.289, 1.504; TyG-WHtR: OR = 1.839, 95% CI = 1.621, 2.087; TyG-CI: OR = 1.233, 95% CI = 1.168, 1.301; TyG-WWI: OR = 1.027, 95% CI = 1.020, 1.033; TyG-BRI: OR = 1.029, 95% CI = 1.022, 1.037). A linear relationship was observed between TyG index and CAD risk (*P*-nonlinear = 0.053). A nonlinear relationship was found between TyG-related indices in relation to CAD risk (*P*-nonlinear ≤ 0.001). Threshold effect analysis showed that after surpassing a certain threshold, the association between TyG-related indices and CAD risk became more significant. ROC analysis revealed that TyG-WHtR had similar predictive ability to the TyG index, while other combinations had lower predictive efficacy than the TyG index alone. Subgroup analyses across different strata consistently demonstrated significant associations between TyG index and its related indices and CAD risk. Mediation analysis showed that HbA1c partially mediated the relationship between TyG index and its related indices with CAD risk, with the highest contribution rate being 27.86%.

**Conclusion:**

The TyG index and its related indices are significantly associated with the risk of CAD, and HbA1c partially mediates this relationship. Furthermore, the clinical applicability of TyG-related indices need further validation to avoid over-interpretation.

## Introduction

Cardiovascular diseases (CVD), particularly stroke and ischemic heart disease, remain major threats to global public health, with their overall burden continuing to rise. In 2019, the number of new CVD cases worldwide exceeded 500 million, approximately double the number reported in 1990 (1). According to data from the World Health Organization (WHO), CVDs account for approximately 17.9 million deaths annually, with coronary artery disease (CAD) being a significant contributor. As the global population ages and lifestyles change, the incidence of CAD has continued to rise, placing immense pressure on healthcare systems worldwide. The medical costs and socioeconomic burden of CAD are exceptionally high. A multinational study involving 22 countries and millions of people found that the annual direct treatment costs for CAD patients account for an average of approximately 21.7% of the per capita GDP in the respective countries. In some countries, this cost even exceeds 100% of the per capita GDP (2). Particularly in low- and middle-income countries, where overall healthcare spending is lower than in high-income countries, the proportion of GDP spent on CVD treatment is even higher due to limited economic capacity, further exacerbating economic pressures in these regions (3). Therefore, early identification and intervention for high-risk CAD patients can not only effectively reduce adverse outcomes but also significantly alleviate the economic burden on healthcare systems, thus providing important public health value and cost-effectiveness.

The occurrence and progression of CAD are not only influenced by traditional risk factors such as hypertension (HTN), dyslipidemia, and smoking, but are also closely related to metabolic abnormalities, particularly insulin resistance (IR). IR is characterized by a weakened response of tissues to insulin, leading to impaired glucose uptake, hyperglycemia, and various metabolic disorders (4). IR impairs insulin signaling in vascular endothelial cells and smooth muscle cells, affecting nitric oxide (NO) production, causing decreased endothelial function, promoting oxidative stress, and inducing low-grade chronic inflammation, thereby driving the formation and instability of atherosclerotic plaques (5, 6). According to Di Pino’s review (7), IR has an “independent and interactive” relationship with atherosclerosis. Additionally, a Mendelian randomization-based proteomic study has found (8) that genetically predicted fasting insulin levels are significantly associated with CAD risk (OR = 1.79, 95% CI = 1.34, 2.40) and identified five proteins as potential mediators of insulin signaling to CAD. This study reinforces that IR is not only a metabolic phenomenon but also a mechanistic foundation influencing coronary artery lesions through specific molecular/protein networks. However, traditional methods for assessing IR, such as hyperinsulinemic euglycemic clamps, are complex, costly, and difficult to implement in large-scale population screening. In recent years, studies have proposed using the easily accessible triglyceride-glucose (TyG) index as a surrogate marker for IR. This index has shown good sensitivity and specificity in clinical and epidemiological studies and is involved in the prediction of metabolic syndrome, HTN, and cardiovascular disease risk (9, 10).

In a U.S. study targeting individuals with diabetes mellitus (DM) or prediabetes (11), the TyG index showed a linear association with the occurrence of CVD, with the highest quartile group exhibiting a significantly higher CVD incidence compared to the lowest group (13.65% vs 7.35%). In a longitudinal cohort study of middle-aged and elderly individuals in China (12), the combination of the TyG index and high-sensitivity C-reactive protein (hsCRP) demonstrated incremental predictive value for cardiovascular events. Compared to the low TyG + low hsCRP group, the high TyG + high hsCRP group had an approximately 30% increased adjusted risk of cardiovascular events. Furthermore, in a population with chronic kidney disease (CKD), a significant positive correlation between the TyG index and CAD risk was observed (OR = 1.617, 95% CI = 1.123, 2.327), with a stronger association in higher stages of CKD (13). Although the TyG index has been established as an independent predictor of CVD risk, using the TyG index alone may not fully capture the additional risk posed by obesity or fat distribution. As a result, researchers have combined the TyG index with various obesity-related indicators, including body mass index (BMI), waist circumference (WC), waist-to-height ratio (WHtR), conicity index (CI), weight-adjusted waist index (WWI), and body roundness index (BRI), to explore their role in predicting cardiovascular event risk. The introduction of these combined indices aims to construct a more comprehensive metabolic-obesity risk assessment framework by simultaneously reflecting IR (as represented by TyG index) and obesity distribution (as represented by the above obesity indicators), thereby enhancing early detection capabilities for cardiovascular events. Although previous studies have indicated that the TyG-related indices is significantly associated with CVD and its mortality risk in certain populations, an analysis based on U.S. NHANES data (14) showed that, while the combination of TyG index and obesity indicators was significantly associated with CVD risk, it did not clearly demonstrate that all combined indices outperformed the TyG index when used alone in terms of predictive performance. Therefore, the aim of this study is to assess the potential value of the TyG index and its combinations with obesity indicators (such as TyG-BMI, TyG-WC, TyG-WHtR, TyG-CI, TyG-WWI, and TyG-BRI) in predicting CAD risk, rather than presuming that these combined indices will necessarily outperform the TyG index alone. This study aims to validate the “significance” of these combined indices and provide empirical evidence for future research, avoiding overgeneralization.

## Methods

### Study population

This study retrospectively assessed the admission data of 18, 897 patients who underwent coronary angiography (CAG) for the first time at Cangzhou People’s Hospital between January 2023 and September 2025. To enhance the robustness of the study, participants were excluded based on predetermined exclusion criteria: (1) patients with a previous diagnosis of CAD or those admitted due to acute myocardial infarction (N = 4, 416); (2) patients lacking data on triglycerides (TG), fasting blood glucose (FBG), weight, height, or WC, preventing the calculation of the TyG index and its related indices (N = 10, 127); (3) patients without baseline covariate data (N = 713). After excluding these factors, a total of 3, 641 participants were included for further analysis. The primary aim of this study was to investigate the comprehensive index of TyG and its associated obesity-related indices and assess their potential association with the risk of CAD. The study adhered to the ethical guidelines of the Declaration of Helsinki and was approved by the Ethics Committee of Cangzhou People’s Hospital (Approval No: K2025-130-02), with a waiver of informed consent.

### Covariates and definitions of TyG index and its related indices

The data for this study were sourced from an electronic health record system, which included participants’ key demographic characteristics, medical history, blood analysis results, and relevant medical imaging records. Demographic characteristics included age, gender, weight (kg), height (cm), BMI (kg/m²), WC (cm), and smoking and drinking status. Clinical history included heart failure (HF), atrial fibrillation (AF), chronic obstructive pulmonary disease (COPD), HTN, and DM.

Smoking was defined as current smoking or a history of smoking, and drinking was defined as current drinking or a history of drinking. The definition of AF included any of the following criteria: physician-diagnosed AF or electrocardiogram (ECG)-confirmed AF. The definition of HF included any of the following: physician-diagnosed HF or cardiac imaging or biomarkers (such as elevated brain natriuretic peptide BNP or NT-proBNP) indicating congestion or impaired pumping function. COPD was defined by any of the following criteria: physician-diagnosed COPD, or pulmonary function tests or imaging showing airflow limitation (e.g., post-bronchodilator FEV_1_/FVC < 0.70) along with typical symptoms (e.g., chronic cough, excessive phlegm, shortness of breath), and/or a history of smoking or other hazardous exposures. HTN was defined by any of the following: systolic blood pressure ≥ 140 mmHg (average of three consecutive measurements), diastolic blood pressure ≥ 90 mmHg (average of three consecutive measurements), or current use of antihypertensive medication, or physician-diagnosed HTN. DM was defined by meeting at least one of the following criteria: FBG ≥ 126 mg/dL, glycated hemoglobin (HbA1c) ≥ 6.5%, current use of antidiabetic medication, or physician-diagnosed DM. Blood analysis data were collected through fasting venous blood samples, with measurements including low-density lipoprotein cholesterol (LDL-C, mmol/L), high-density lipoprotein cholesterol (HDL-C, mmol/L), TG (mmol/L), total cholesterol (TC, mmol/L), FBG (mmol/L), HbA1c (%), alanine aminotransferase (ALT, U/L), aspartate aminotransferase (AST, U/L), serum creatinine (SCr, μmol/L), cystatin C (Cystatin C, mg/L), and uric acid (SUA, μmol/L). The diagnosis of CAD was established through CAG, performed via percutaneous radial or femoral artery catheterization.

To ensure consistency in unit conversion, FBG and TG were converted from mmol/L to mg/dL using the following factors: FBG (mg/dL) = FBG (mmol/L)×18.016; TG (mg/dL) = TG (mmol/L)×88.57. The TyG index was then calculated as:


TyG=ln [TG(mg / dL)×FBG (mg/dL) / 2]

Based on previous studies (15–17), the following obesity indices were derived. Where applicable, height was converted from cm to m, and WC was converted from cm to m to ensure dimensional consistency in the formulas:


BMI=Weight (kg) / Height (m2)2;

WHtR=WC (cm) / Height (cm);

$$\text{CI}=0.109^{-1}\times \text{WC}(\text{m})\times {\left[\text{Weight}\;(\text{kg})\;/\;\text{Height}\;(\text{m})\right]}^{-1/2};$$

$$\text{WWI}=\text{WC}\;(\text{cm})\times \text{Weight}{\;(\text{kg})}^{-1/2};$$

$$\text{BRI}=364.2-365.5\times {\left(1-{\left[\text{WC}\;(\text{cm})\;/\;2\text{π}\right]}^{2}\;/{\;[0.5\times \text{Height}\;(\text{cm})]}^{2}\right)}^{-1/2}.$$

The TyG-related combined indices were then computed as the product of the TyG index and each obesity indicator:


TyG−BMI=TyG×BMI;

TyG−WC=TyG×WC;

TyG−WHtR=TyG×WHtR;

TyG−CI=TyG×CI;

TyG−WWI=TyG×WWI;

TyG−BRI=TyG×BRI.

All indices were treated as continuous variables in the statistical analyses.

### Definitions of CAD

CAD is defined as the narrowing or blockage of the coronary artery lumen due to the accumulation of fat, cholesterol, and other substances, which restricts blood supply to the myocardium. The characteristic of CAD is a ≥50% stenosis of a single major coronary artery lumen (18).

### Statistical analysis

The Kolmogorov-Smirnov (KS) test is used to assess the normality of continuous variables. For continuous variables that do not follow a normal distribution, the median and interquartile range (IQR) are used for description, while categorical variables are presented as frequencies (N, %). Subsequently, the Mann-Whitney U test (for continuous variables that do not follow a normal distribution) or Pearson’s chi-square test (for categorical variables) is used to compare baseline characteristics between the two groups. We constructed univariate and multivariate logistic regression models to evaluate the association between the TyG index and its related indices with CAD risk, with results presented as odds ratios (OR) and 95% confidence intervals (95% CI). Multicollinearity in the logistic regression models was assessed using the variance inflation factor (VIF), and all variables had a VIF of less than 5, indicating no significant multicollinearity. Model 1 was unadjusted for covariates, while Model 2 adjusted for age, gender, COPD, HTN, DM, smoking status, HDL-C, LDL-C, HbA1c, SCr, cystatin C, and SUA. Additionally, we performed restricted cubic spline (RCS) regression for the four percentiles (5th, 35th, 65th, and 95th) of the TyG index and its related indices to test for nonlinear associations and describe the dose-response relationship between the TyG index and CAD risk. If a nonlinear relationship exists, we estimate the turning points by maximizing the goodness of fit and select the optimal threshold. We then use a two-segment logistic regression model to assess the threshold effect of TyG-related indices on CAD risk. Turning points are determined using a recursive algorithm, and the goodness of fit between the linear model and the segmented model is compared using the likelihood ratio test. We compared the classification performance of the logistic regression model and the segmented regression model in predicting CAD. The predictive ability of the models was assessed by calculating the Area Under the Receiver Operating Characteristic (ROC) Curve (AUC) and comparing the differences in AUC between the two models using the DeLong test, with the optimal model selected.

To further assess the discriminatory power of the TyG index and its related indices in predicting CAD risk, we performed Receiver Operating Characteristic (ROC) curve analysis and compared the differences in AUC between the indicators using DeLong’s test. Additionally, subgroup analyses were performed based on baseline age (<60 years vs. ≥60 years), gender (male vs. female), smoking status (smoker vs. non-smoker), BMI (<25 kg/m² vs. ≥25 kg/m²), HTN (present vs. absent), and DM (present vs. absent) to evaluate the risk in different susceptible populations. Finally, mediation analysis was conducted to evaluate the total and direct effects of TyG index and its related indicators on CAD, and to analyze the mediating role of HbA1c. The average causal mediation effect (ACME), average direct effect (ADE), and the proportion mediated were calculated to assess the indirect impact. All statistical analyses were conducted using R software version 4.3.3 (Vienna, Austria). A two-tailed P value less than 0.05 was regarded as statistically significant.

## Results

### Participant characteristics

A total of 3, 641 participants were included in this study, with 52.51% being male, and the mean age was 63.00 (14.00) years. Participants were divided into the non-CAD group (N = 1, 885) and the CAD group (N = 1, 756) based on the presence of CAD. **Table 1** summarizes the baseline characteristics of the participants. There were significant differences between the two groups in terms of age, gender, BMI, WC, WHtR, CI, WWI, BRI, TyG and its combinations with obesity indicators (TyG-BMI, TyG-WC, TyG-WHtR, TyG-CI, TyG-WWI, TyG-BRI), as well as smoking, COPD, HTN, and DM prevalence (all *P* < 0.05). Additionally, there were significant differences in laboratory markers, including LDL-C, HDL-C, TG, TC, FBG, HbA1c, SCr, Cystatin C, and SUA (all *P* < 0.05).

**Table 1:** Baseline characteristics stratified by the occurrence of CAD.

Variables	All participants	Non-CAD	CAD	*P* value
Age (years, median, IQR)	63.00 (14.00)	61.00 (14.00)	64.00 (14.00)	< 0.001
Males (N, %)	1, 912.00 (52.51%)	906.00 (48.06%)	1, 006.00 (57.29%)	< 0.001
HF (N, %)	373.00 (10.24%)	178.00 (9.44%)	195.00 (11.10%)	0.098
AF (N, %)	76.00 (2.09%)	35.00 (1.86%)	41.00 (2.33%)	0.313
COPD (N, %)	147.00 (4.04%)	56.00 (2.97%)	91.00 (5.18%)	< 0.001
HTN (N, %)	2, 331.00 (64.02%)	1, 158.00 (61.43%)	1, 173.00 (66.80%)	< 0.001
DM (N, %)	716.00 (19.66%)	327.00 (17.35%)	389.00 (22.15%)	< 0.001
Smoking (N, %)	761.00 (20.90%)	345.00 (18.30%)	416.00 (23.69%)	< 0.001
Drinking (N, %)	681.00 (18.70%)	332.00 (17.61%)	349.00 (19.87%)	0.080
BMI (kg/m^2^, median, IQR)	24.91 (3.23)	24.81 (3.07)	25.01 (3.58)	0.029
WC (cm, median, IQR)	85.00 (12.00)	85.00 (11.00)	86.00 (13.00)	< 0.001
WHtR (median, IQR)	0.51 (0.07)	0.51 (0.06)	0.52 (0.08)	< 0.001
CI (median, IQR)	1.23 (0.16)	1.22 (0.15)	1.23 (0.16)	0.005
WWI (median, IQR)	10.38 (1.38)	10.33 (1.30)	10.43 (1.43)	< 0.001
BRI (median, IQR)	3.59 (1.33)	3.53 (1.25)	3.65 (1.50)	< 0.001
LDL-C (mmol/L, median, IQR)	2.66 (1.30)	2.59 (1.23)	2.76 (1.31)	< 0.001
HDL-C (mmol/L, median, IQR)	1.18 (0.40)	1.22 (0.39)	1.13 (0.40)	< 0.001
TG (mmol/L, median, IQR)	1.39 (1.02)	1.27 (0.86)	1.55 (1.14)	< 0.001
TC (mmol/L, median, IQR)	4.42 (1.51)	4.34 (1.42)	4.52 (1.64)	< 0.001
FBG (mmol/L, median, IQR)	5.88 (2.21)	5.66 (1.70)	6.24 (2.95)	< 0.001
HbA1c (%, median, IQR)	6.10 (1.40)	6.00 (1.00)	6.30 (1.80)	< 0.001
ALT (U/L, median, IQR)	19.00 (13.00)	18.00 (12.00)	19.00 (13.00)	0.260
AST (U/L, median, IQR)	20.00 (8.00)	20.00 (7.00)	19.00 (8.00)	0.449
SCr (μmol/L, median, IQR)	63.00 (21.70)	62.00 (20.00)	65.00 (22.00)	< 0.001
Cystatin C (mg/L, median, IQR)	0.95 (0.33)	0.93 (0.30)	0.98 (0.34)	< 0.001
SUA (μmol/L, median, IQR)	295.00 (116.00)	289.00 (111.00)	302.00 (122.00)	< 0.001
TyG (median, IQR)	8.84 (0.86)	8.70 (0.78)	9.00 (0.94)	< 0.001
TyG-BMI (median, IQR)	220.73 (37.93)	216.64 (34.45)	227.36 (40.51)	< 0.001
TyG-WC (median, IQR)	7.62 (1.29)	7.46 (1.17)	7.81 (1.44)	< 0.001
TyG-WHtR (median, IQR)	4.56 (0.79)	4.45 (0.70)	4.68 (0.89)	< 0.001
TyG-CI (median, IQR)	10.92 (1.84)	10.69 (1.64)	11.17 (2.01)	< 0.001
TyG-WWI (median, IQR)	91.98 (15.73)	90.00 (13.84)	94.50 (17.13)	< 0.001
TyG-BRI (median, IQR)	31.95 (12.22)	31.03 (10.76)	33.20 (14.27)	< 0.001

CAD, Coronary Artery Disease; IQR, Interquartile Range; HF, Heart Failure; AF, Atrial Fibrillation; COPD, Chronic Obstructive Pulmonary Disease; HTN; Hypertension; DM, Diabetes Mellitus; BMI, Body Mass Index; WC, Waist Circumference; WHtR, Waist-to-Height Ratio; CI, Conicity Index; WWI, Weight-adjusted Waist Index; BRI, Body Roundness Index, LDL-C, Low-Density Lipoprotein Cholesterol, HDL-C, High-Density Lipoprotein Cholesterol; TG; Triglycerides; TC, Total Cholesterol; FBG, Fasting Blood Glucose; HbA1c, Glycated Hemoglobin; ALT, Alanine Aminotransferase; AST, Aspartate Aminotransferase; SCr, Serum Creatinine; SUA, Serum Uric Acid; TyG, Triglyceride-Glucose Index.

### Association between TyG index and its related indices and the risk of CAD occurrence

Prior to performing logistic regression analysis, we calculated the VIFs for all covariates, setting a threshold of 5. A VIF above 5 was considered indicative of significant multicollinearity. The results showed that all variables included in the multivariate logistic regression analysis had VIFs below 5, indicating no significant multicollinearity.

After adjusting for covariates, the logistic regression analysis results in **Table 2** showed that for each 1 standard deviation increase in the TyG index and its related indices, the risk of CAD increased by 64.2% (TyG, OR = 1.642, 95% CI = 1.447, 1.866), 1.0% (TyG-BMI, OR = 1.010, 95% CI: 1.007, 1.012), 39.2% (TyG-WC, OR = 1.392, 95% CI = 1.289, 1.504), 83.9% (TyG-WHtR, OR = 1.839, 95% CI = 1.621, 2.087), 23.3% (TyG-CI, OR = 1.233, 95% CI = 1.168, 1.301), 2.7% (TyG-WWI, OR = 1.027, 95% CI = 1.020, 1.033), and 2.9% (TyG-BRI, OR = 1.029, 95% CI = 1.022, 1.037). Compared to the lowest quartile, the OR (95% CI) for CAD occurrence in the fourth quartile was as follows: 1.881 (95% CI = 1.495, 2.368) for TyG, 1.894 (95% CI = 1.545, 2.325) for TyG-BMI, 2.044 (95% CI = 1.664, 2.513) for TyG-WC, 2.144 (95% CI = 1.745, 2.635) for TyG-WHtR, 1.791 (95% CI = 1.458, 2.202) for TyG-CI, 1.985 (95% CI = 1.615, 2.442) for TyG-WWI, and 1.759 (95% CI = 1.445, 2.144) for TyG-BRI.

**Table 2:** Association between TyG index and its related indices and the risk of CAD occurrence.

CAD	Model 1	*P* value	Model 2	*P* value
	OR (95% CI)		OR (95% CI)	
TyG	2.188 (1.977, 2.431)	< 0.001	1.642 (1.447, 1.866)	< 0.001
Q1	Ref.		Ref.	
Q2	1.302 (1.077, 1.574)	0.007	1.061 (0.869, 1.295)	0.561
Q3	2.064 (1.711, 2.494)	< 0.001	1.458 (1.186, 1.793)	< 0.001
Q4	3.438 (2.839, 4.172)	< 0.001	1.881 (1.495, 2.368)	< 0.001
TyG-BMI	1.014 (1.012, 1.017)	< 0.001	1.010 (1.007, 1.012)	< 0.001
Q1	Ref.		Ref.	
Q2	1.235 (1.024, 1.489)	0.027	1.088 (0.895, 1.324)	0.397
Q3	1.520 (1.262, 1.833)	< 0.001	1.193 (0.979, 1.454)	0.081
Q4	2.650 (2.194, 3.204)	< 0.001	1.894 (1.545, 2.325)	< 0.001
TyG-WC	1.606 (1.497, 1.725)	< 0.001	1.392 (1.289, 1.504)	< 0.001
Q1	Ref.		Ref.	
Q2	1.417 (1.175, 1.710)	< 0.001	1.199 (0.985, 1.459)	0.070
Q3	1.575 (1.307, 1.900)	< 0.001	1.236 (1.014, 1.508)	0.036
Q4	3.008 (2.487, 3.643)	< 0.001	2.044 (1.664, 2.513)	< 0.001
TyG-WHtR	2.265 (2.017, 2.548)	< 0.001	1.839 (1.621, 2.087)	< 0.001
Q1	Ref.		Ref.	< 0.001
Q2	1.266 (1.049, 1.528)	0.014	1.108 (0.910, 1.349)	0.308
Q3	1.637 (1.358, 1.974)	< 0.001	1.271 (1.042, 1.550)	0.018
Q4	3.008 (2.488, 3.643)	< 0.001	2.144 (1.745, 2.635)	
TyG-CI	1.372 (1.307, 1.443)	< 0.001	1.233 (1.168, 1.301)	< 0.001
Q1	Ref.		Ref.	
Q2	1.164 (0.966, 1.404)	0.111	0.972 (0.799, 1.183)	0.776
Q3	1.441 (1.196, 1.756)	< 0.001	1.102 (0.905, 1.343)	0.333
Q4	2.714 (2.247, 3.283)	< 0.001	1.791 (1.458, 2.202)	< 0.001
TyG-WWI	1.039 (1.033, 1.045)	< 0.001	1.027 (1.020, 1.033)	< 0.001
Q1	Ref.		Ref.	< 0.001
Q2	1.223 (1.015, 1.475)	0.035	1.054 (0.866, 1.283)	0.602
Q3	1.313 (1.090, 1.583)	0.004	1.018 (0.835, 1.241)	0.860
Q4	2.901 (2.400, 3.513)	< 0.001	1.985 (1.615, 2.442)	
TyG-BRI	1.034 (1.027, 1.041)	< 0.001	1.029 (1.022, 1.037)	< 0.001
Q1	Ref.		Ref.	
Q2	1.081 (0.898, 1.301)	0.410	1.040 (0.856, 1.265)	0.690
Q3	1.140 (0.948, 1.372)	0.165	1.052 (0.865, 1.280)	0.609
Q4	2.022 (1.678, 2.438)	< 0.001	1.759 (1.445, 2.144)	< 0.001

Model 1: unadjusted model.

Model 2: adjusted for age, gender, COPD, HTN, DM, Smoking, HDL-C, LDL-C, HbA1c, SCr, Cystatin C, and SUA

OR, Odds Ratio; CI, Confidence Interval; CAD, Coronary Artery Disease; Q; Quartiles; COPD, Chronic Obstructive Pulmonary Disease; HTN; Hypertension; DM, Diabetes Mellitus, HDL-C, High-Density Lipoprotein Cholesterol, LDL-C, Low-Density Lipoprotein Cholesterol; HbA1c, Glycated hemoglobin; SCr, Serum Creatinine; SUA, Serum Uric Acid.

Overall, the TyG index and its related indices were strongly associated with the risk of CAD, underscoring their potential as cardiovascular disease risk markers.

**Figure 1:**
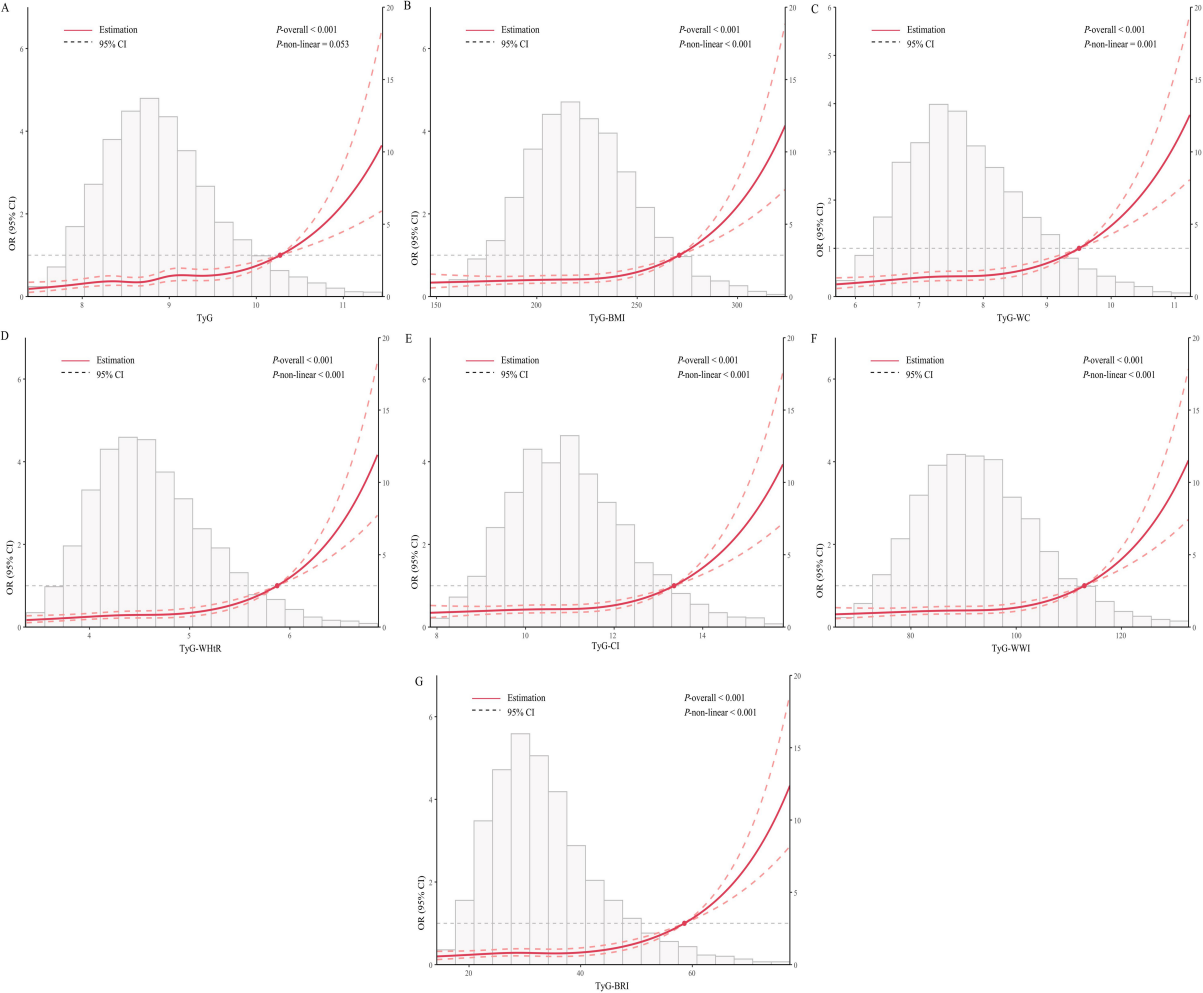
Dose-response relationship between TyG index and its related indices and the risk of CAD occurrence. adjusted for age, gender, COPD, HTN, DM, Smoking, HDL-C, LDL-C, HbA1c, SCr, Cystatin C, and SUA. **(A)** Association between TyG and CAD. **(B)** Association between TyG-BMI and CAD. **(C)** Association between TyG-WC and CAD. **(D)** Association between TyG-WHtR and CAD. **(E)** Association between TyG-CI and CAD. **(F)** Association between TyG-WWI and CAD. **(G)** Association between TyG-BRI and CAD. OR, Odds Ratio; CI, Confidence Interval; CAD, Coronary Artery Disease; Q, Quartiles; COPD, Chronic Obstructive Pulmonary Disease; HTN, Hypertension; DM, Diabetes Mellitus, HDL-C, High-Density Lipoprotein Cholesterol, LDL-C, Low-Density Lipoprotein Cholesterol; HbA1c, Glycated hemoglobin; SCr, Serum Creatinine; SUA, Serum Uric Acid.

After comprehensive adjustment for covariates, the RCS regression analysis revealed a dose-response relationship between the TyG index and its related indices and the incidence of CAD. The TyG showed a linear relationship with overall CAD incidence (*P*-nonlinear = 0.053, **Figure 1A**). Furthermore, TyG-BMI, TyG-WC, TyG-WHtR, TyG-CI, TyG-WWI, and TyG-BRI demonstrated a nonlinear dose-response relationship with overall CAD incidence (*P*-nonlinear ≤ 0.001, **Figures 1B-D, F, G**).

### The relationship between the TyG-related indices and CAD risk using threshold effect analysis

According to **Table 3**, for TyG-BMI, each 1 standard deviation increase above the inflection point was associated with a 2.6% increase in CAD risk (OR = 1.026, 95% CI = 1.019, 1.034, *P* < 0.001). Below the inflection point, however, the association between TyG-BMI and CAD risk was not significant (OR = 1.003, 95% CI = 1.000, 1.007, *P* = 0.064). For TyG-CI, each 1 standard deviation increase above the inflection point was associated with a 70.1% increase in CAD risk (OR = 1.701, 95% CI = 1.458, 1.985, *P* < 0.001). Below the inflection point, no significant association between TyG-CI and CAD risk was observed (OR = 1.076, 95% CI = 0.996, 1.164, *P* = 0.064). For TyG-WWI, each 1 standard deviation increase above the inflection point was associated with a 6.0% increase in CAD risk (OR = 1.060, 95% CI = 1.044, 1.075, *P* < 0.001). Below the inflection point, the association between TyG-WWI and CAD risk was not significant (OR = 1.006, 95% CI = 0.996, 1.016, *P* = 0.261).

**Table 3:** The relationship between TyG-related indices with CAD risk using threshold effect analysis.

Variables	Adjusted OR (95% CI)	*P* value
CAD		
TyG-BMI		
Logistic regression model	1.010 (1.007, 1.012)	< 0.001
Segmented regression model		
Inflection point	241.329	
< 241.329	1.003 (1.000, 1.007)	0.064
> 241.329	1.026 (1.019, 1.034)	< 0.001
Log-likelihood ratio		< 0.001
TyG-WC		
Logistic regression model	1.392 (1.289, 1.504)	< 0.001
Segmented regression model		
Inflection point	8.547	
< 8.547	1.202 (1.079, 1.339)	< 0.001
> 8.547	2.114 (1.657, 2.696)	< 0.001
Log-likelihood ratio		< 0.001
TyG-WHtR		
Logistic regression model	1.839 (1.621, 2.087)	< 0.001
Segmented regression model		
Inflection point	5.099	
< 5.099	1.364 (1.139, 1.635)	< 0.001
> 5.099	2.185 (1.743, 2.840)	< 0.001
Log-likelihood ratio		< 0.001
TyG-CI		
Logistic regression model	1.233 (1.168, 1.301)	< 0.001
Segmented regression model		
Inflection point	11.928	
< 11.928	1.076 (0.996, 1.164)	0.064
> 11.928	1.701 (1.458, 1.985)	< 0.001
Log-likelihood ratio		< 0.001
TyG-WWI		
Logistic regression model	1.027 (1.020, 1.033)	< 0.001
Segmented regression model		
Inflection point	97.285	
< 97.285	1.006 (0.996, 1.016)	0.261
> 97.285	1.060 (1.044, 1.075)	< 0.001
Log-likelihood ratio		< 0.001
TyG-BRI		
Logistic regression model	1.029 (1.022, 1.037)	< 0.001
Segmented regression model		
Inflection point	48.051	
< 48.051	1.014 (1.005, 1.023)	0.003
> 48.051	1.107 (1.072, 1.144)	< 0.001
Log-likelihood ratio		< 0.001

adjusted for age, gender, COPD, HTN, DM, Smoke, HDL-C, LDL-C, HbA1c, SCr, Cystatin C, and SUA.

OR, Odds Ratio; CI, Confidence Interval; CAD, Coronary Artery Disease; Q; Quartiles; COPD, Chronic Obstructive Pulmonary Disease; HTN; Hypertension; DM, Diabetes Mellitus, HDL-C, High-Density Lipoprotein Cholesterol, LDL-C, Low-Density Lipoprotein Cholesterol; HbA1c, Glycated hemoglobin; SCr, Serum Creatinine; SUA, Serum Uric Acid.

Additionally, for TyG-WC, each 1 standard deviation increase above the inflection point was associated with a 111.4% increase in CAD risk (OR = 2.114, 95% CI = 1.657, 2.696, *P* < 0.001). Below the inflection point, the association with CAD risk was still statistically significant but lower (OR = 1.202, 95% CI = 1.079, 1.339, *P* < 0.001). For TyG-WHtR, each 1 standard deviation increase above the inflection point was associated with a 118.5% increase in CAD risk (OR = 2.185, 95% CI = 1.743, 2.840, *P* < 0.001). Below the inflection point, the association with CAD risk remained significant, though the increase was smaller (OR = 1.364, 95% CI = 1.139, 1.635, *P* < 0.001). For TyG-BRI, each 1 standard deviation increase above the inflection point was associated with a 10.7% increase in CAD risk (OR = 1.107, 95% CI = 1.072, 1.144, *P* < 0.001). Below the inflection point, the association was still significant but weaker (OR = 1.014, 95% CI = 1.005, 1.023, *P* = 0.003).

**Figure 2:**
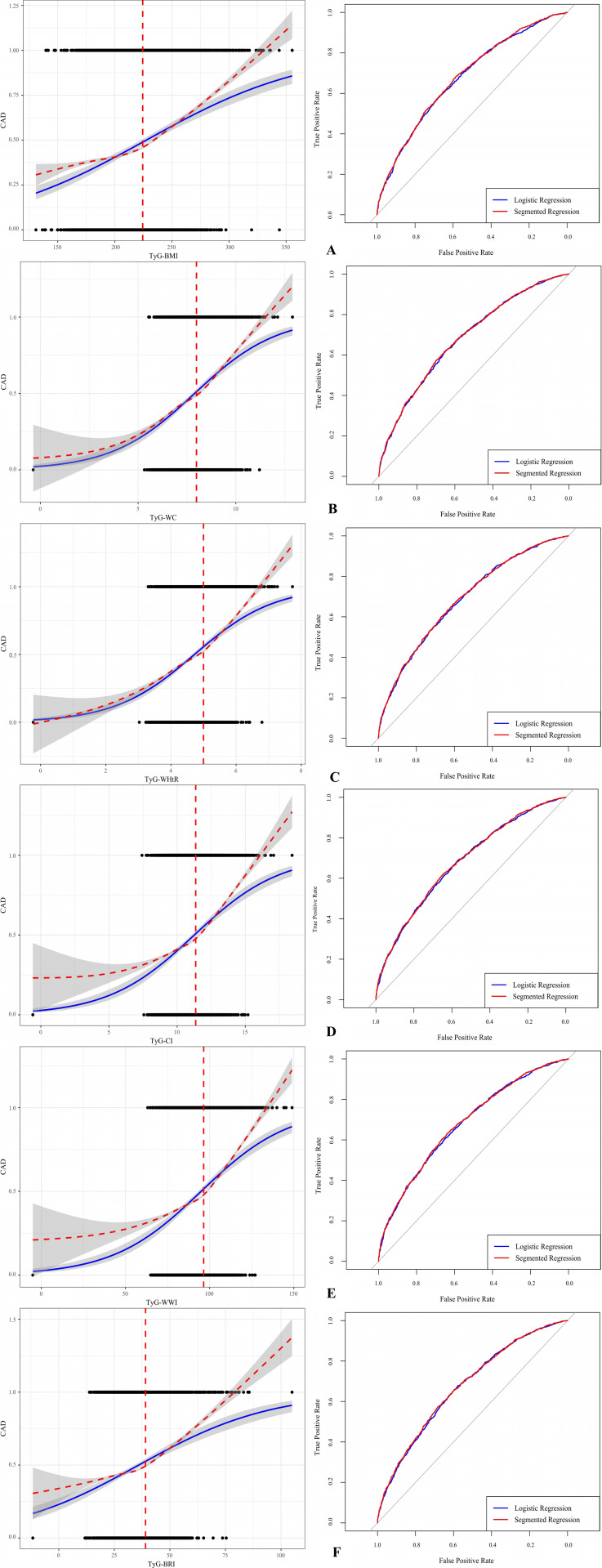
Comparison of Logistic and Segmented Regression Models. Adjusted for age, gender, COPD, HTN, DM, Smoking, HDL-C, LDL-C, HbA1c, SCr, Cystatin C, and SUA. **(A)** TyG-BMI Models Comparison. **(B)** TyG-WC Models Comparison. **(C)** TyG-WHtR Models Comparison. **(D)** TyG-CI Models Comparison. **(E)** TyG-WWI Models Comparison. **(F)** TyG-BRI Models Comparison. OR, Odds Ratio; CI, Confidence Interval; CAD, Coronary Artery Disease; Q, Quartiles; COPD, Chronic Obstructive Pulmonary Disease; HTN, Hypertension; DM, Diabetes Mellitus, HDL-C, High-Density Lipoprotein Cholesterol, LDL-C, Low-Density Lipoprotein Cholesterol; HbA1c, Glycated hemoglobin; SCr, Serum Creatinine; SUA, Serum Uric Acid.

**Figure 2** compare the logistic regression model and the segmented regression model to evaluate the classification performance of the TyG-related indices in predicting CAD. The results showed that the prediction performance of TyG-BMI (*P* = 0.064), TyG-WC (*P* = 0.086), TyG-CI (*P* = 0.075), TyG-WWI, and TyG-BRI (*P* = 0.053) did not differ significantly between the two models. However, TyG-WHtR (*P* = 0.019) and TyG-WWI (*P* = 0.036) showed significant differences in prediction performance between the two models, with the segmented regression model performing slightly better than the logistic regression model.

### Prognostic performance of the TyG index and its related indices for CAD risk

**Figure 3:**
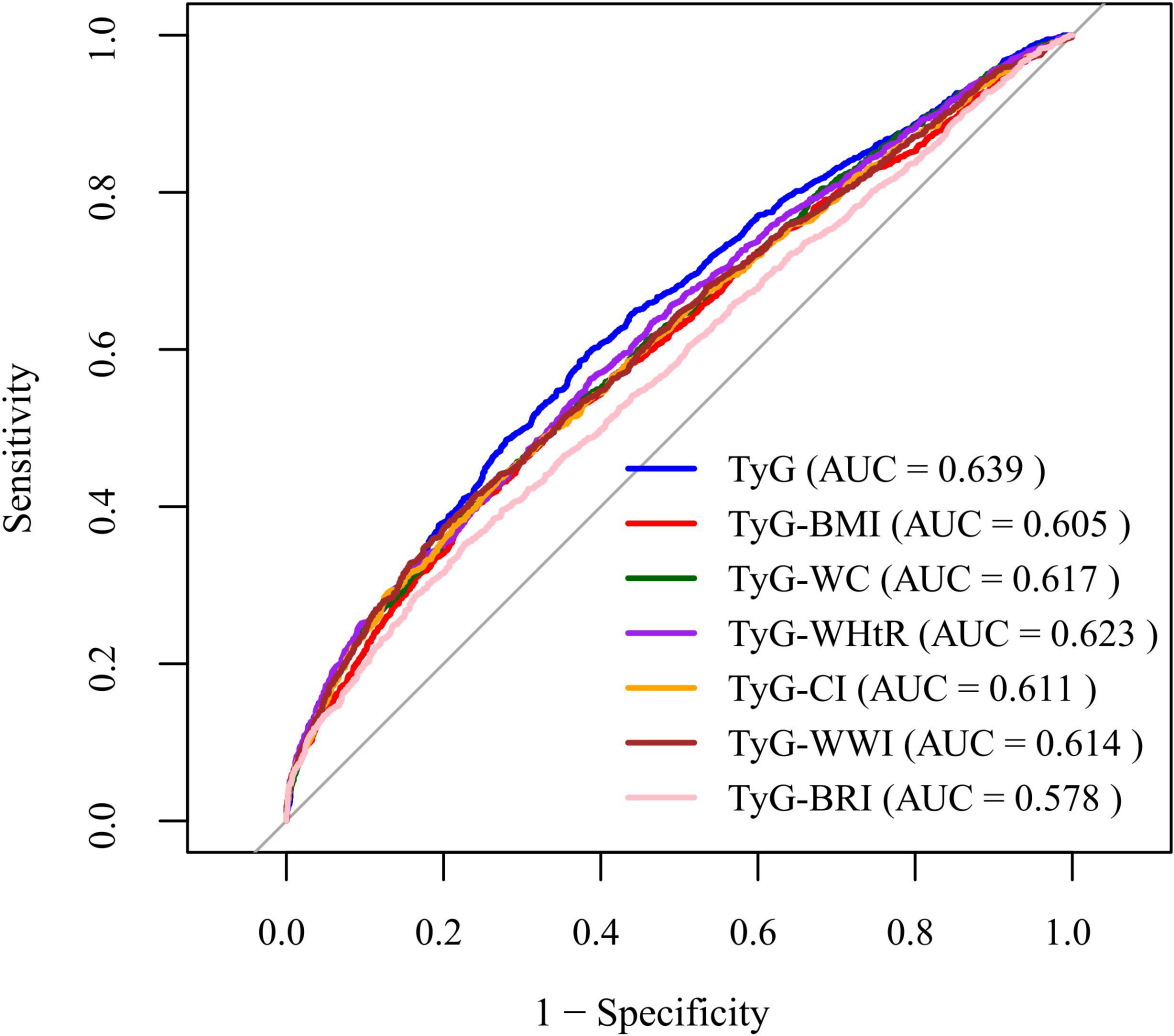
ROC curve for the TyG index and its related indices.

In the ROC curve analysis, the predictive value of the TyG index and its related indices for CAD is shown in **Figure 3** and **Table 4**. The specific results are as follows: the AUC for TyG predicting CAD risk was 0.639 (95% CI = 0.623, 0.654); TyG-BMI (AUC = 0.605, 95% CI = 0.589, 0.621); TyG-WC (AUC = 0.617, 95% CI = 0.601, 0.632); TyG-WHtR (AUC = 0.623, 95% CI = 0.607, 0.638); TyG-CI (AUC = 0.611, 95% CI = 0.595, 0.627); TyG-WWI (AUC = 0.614, 95% CI = 0.598, 0.630); TyG-BRI (AUC = 0.578, 95% CI = 0.562, 0.594). These results indicate that the TyG index and its related indices have some discriminative ability in predicting the risk of CAD. The DeLong test results, shown in **Table 5**, revealed statistically significant differences between TyG and TyG-BMI, TyG-WC, TyG-CI, TyG-WWI, TyG-BRI, and between TyG-BRI and TyG-BMI, TyG-WC, TyG-WHtR, TyG-CI, TyG-WWI, and between TyG-WHtR and TyG-CI (all *P* < 0.05). However, other combinations showed differences at the numerical level but did not reach statistical significance (all *P* > 0.05).

**Table 4:** ROC curve for the TyG index and its related indices.

Variables	AUC (95% CI)	Youden index	The optimal cut off value	Sensitivity	Specificity
TyG	0.639 (0.623, 0.654)	0.212	8.860	59.57	61.64
TyG-BMI	0.605 (0.589, 0.621)	0.165	227.311	50.11	66.37
TyG-WC	0.617 (0.601, 0.632)	0.169	7.999	43.91	73.00
TyG-WHtR	0.623 (0.607, 0.638)	0.176	4.601	56.26	61.33
TyG-CI	0.611 (0.595, 0.627)	0.165	11.422	43.68	72.84
TyG-WWI	0.614 (0.598, 0.630)	0.174	97.434	41.46	75.97
TyG-BRI	0.578 (0.562, 0.594)	0.126	37.555	35.31	77.29

AUC, the area under the receiver operating characteristic curve; CI, confidence interval.

**Table 5:** Comparison of AUC values of the TyG index and its related indices (DeLong’s test).

Z, *P* Value	TyG	TyG-BMI	TyG-WC	TyG-WHtR	TyG-CI	TyG-WWI	TyG-BRI
TyG	–	3.744, < 0.001	2.545, 0.011	1.835, 0.067	3.197, 0.001	2.805, 0.005	5.196, < 0.001
TyG-BMI	3.744, < 0.001	–	1.183, 0.237	1.868, 0.062	0.496, 0.620	0.755, 0.450	2.539, 0.011
TyG-WC	2.545, 0.011	1.183, 0.237	–	1.636, 0.102	1.192, 0.233	0.436, 0.663	6.889, < 0.001
TyG-WHtR	1.835, 0.067	1.868, 0.062	1.636, 0.102	–	2.286, 0.022	1.907, 0.057	11.883, < 0.001
TyG-CI	3.197, 0.001	0.496, 0.620	1.192, 0.233	2.286, 0.022	–	1.685, 0.092	4.923, < 0.001
TyG-WWI	2.805, 0.005	0.755, 0.450	0.436, 0.663	1.907, 0.057	1.685, 0.092	–	5.936, < 0.001
TyG-BRI	5.196, < 0.001	2.539, 0.011	6.889, < 0.001	11.883, < 0.001	4.923, < 0.001	5.936, < 0.001	–

AUC, the area under the receiver operating characteristic curve.

### Subgroup analyses

Based on the data in **Table 6**, after adjusting for variables such as age, gender, COPD, HTN, DM, Smoking, HbA1c, HDL-C, LDL-C, SCr, Cystatin C, and SUA, the association between the TyG index and its related indices and CAD risk was evaluated across different subgroups. In the gender subgroup analysis, significant interactions were found for multiple TyG-related indices, indicating that gender significantly moderates these relationships. Specifically, for TyG-WC, the association with CAD was stronger in females (OR = 1.565, 95% CI = 1.397-1.756) than in males (OR = 1.264, 95% CI = 1.137-1.406), with a significant interaction (*P* for interaction = 0.005). Similarly, TyG-WHtR showed a stronger effect in females (OR = 2.222, 95% CI = 1.846-2.686) compared to males (OR = 1.570, 95% CI = 1.323-1.869; *P* for interaction = 0.005). TyG-CI also demonstrated a stronger association in females (OR = 1.327, 95% CI = 1.226-1.439) than in males (OR = 1.154, 95% CI = 1.072-1.245; *P* for interaction = 0.011). For TyG-WWI, the effect was more pronounced in females (OR = 1.036, 95% CI = 1.026-1.045) than in males (OR = 1.019, 95% CI = 1.010-1.028; *P* for interaction = 0.010). Likewise, TyG-BRI exhibited a stronger association in females (OR = 1.039, 95% CI = 1.028-1.049) compared to males (OR = 1.022, 95% CI = 1.012-1.031; *P* for interaction = 0.015). These findings suggest that gender modifies the relationship between TyG-related indices and CAD risk, with consistently stronger associations observed in females.

**Table 6:** Subgroup analyses of the TyG index and its related indices for CAD risk.

Subgroup	N	TyG OR (95% CI) *P* value	TyG-BMI OR (95% CI) *P* value	TyG-WC OR (95% CI) *P* value	TyG-WHtR OR (95% CI) *P* value	TyG-CI OR (95% CI) *P* value	TyG-WWI OR (95% CI) *P* value	TyG-BRI OR (95% CI) *P* value
Age (year)								
< 60	1418	1.410 (1.152, 1.730) < 0.001	1.010 (1.006, 1.014) < 0.001	1.291 (1.139, 1.466) < 0.001	1.637 (1.334, 2.015) < 0.001	1.149 (1.052, 1.255) 0.002	1.018 (1.008, 1.029) < 0.001	1.025 (1.013, 1.037) < 0.001
≥ 60	2223	1.806 (1.533, 2.132) < 0.001	1.010 (1.007, 1.013) < 0.001	1.456 (1.321, 1.608) < 0.001	1.963 (1.673, 2.309) < 0.001	1.283 (1.197, 1.376) < 0.001	1.031 (1.023, 1.040) < 0.001	1.032 (1.023, 1.041) < 0.001
*P* for interaction		0.649	0.703	0.389	0.407	0.220	0.223	0.374
Gender								
Male	1912	1.463 (1.228, 1.746) < 0.001	1.008 (1.005, 1.012) < 0.001	1.264 (1.137, 1.406) < 0.001	1.570 (1.323, 1.869) < 0.001	1.154 (1.072, 1.245) < 0.001	1.019 (1.010, 1.028) < 0.001	1.022 (1.012, 1.031) < 0.001
Female	1729	1.871 (1.557, 2.256) < 0.001	1.012 (1.008, 1.016) < 0.001	1.565 (1.397, 1.756) < 0.001	2.222 (1.846, 2.686) < 0.001	1.327 (1.226, 1.439) < 0.001	1.036 (1.026, 1.045) < 0.001	1.039 (1.028, 1.049) < 0.001
*P* for interaction		0.068	0.100	0.005	0.005	0.011	0.010	0.015
Smoking status								
Yes	761	1.847 (1.390, 2.475) < 0.001	1.011 (1.005, 1.017) < 0.001	1.403 (1.197, 1.653) < 0.001	1.923 (1.472, 2.533) < 0.001	1.249 (1.116, 1.402) < 0.001	1.029 (1.015, 1.043) < 0.001	1.030 (1.016, 1.045) < 0.001
No	2880	1.608 (1.396, 1.856) < 0.001	1.010 (1.007, 1.013) < 0.001	1.388 (1.272, 1.517) < 0.001	1.814 (1.574, 2.096) < 0.001	1.225 (1.151, 1.303) < 0.001	1.026 (1.018, 1.033) < 0.001	1.029 (1.021, 1.037) < 0.001
*P* for interaction		0.908	0.966	0.971	0.922	0.932	0.927	0.872
BMI (kg/m^2^)								
< 25	1852	1.718 (1.439, 2.057) < 0.001	1.008 (1.003, 1.013) 0.002	1.345 (1.203, 1.506) < 0.001	1.758 (1.444, 2.147) < 0.001	1.283 (1.183, 1.394) < 0.001	1.030 (1.021, 1.041) < 0.001	1.025 (1.013, 1.037) < 0.001
≥ 25	1789	1.603 (1.337, 1.926) < 0.001	1.020 (1.015, 1.025) < 0.001	1.425 (1.279, 1.589) < 0.001	1.887 (1.595, 2.240) < 0.001	1.234 (1.144, 1.332) < 0.001	1.027 (1.018, 1.036) < 0.001	1.031 (1.022, 1.041) < 0.001
*P* for interaction		0.213	0.058	0.864	0.977	0.260	0.335	0.532
HTN								
Yes	2331	1.803 (1.538, 2.118) < 0.001	1.010 (1.007, 1.014) < 0.001	1.415 (1.286, 1.559) < 0.001	1.848 (1.582, 2.164) < 0.001	1.245 (1.165, 1.333) < 0.001	1.027 (1.019, 1.035) < 0.001	1.028 (1.019, 1.037) < 0.001
No	1310	1.400 (1.134, 1.733) 0.002	1.009 (1.005, 1.014) < 0.001	1.356 (1.191, 1.546) < 0.001	1.831 (1.479, 2.277) < 0.001	1.206 (1.100, 1.323) < 0.001	1.025 (1.014, 1.037) < 0.001	1.032 (1.020, 1.044) < 0.001
*P* for interaction		0.221	0.696	0.740	0.955	0.730	0.918	0.652
DM								
Yes	716	1.640 (1.237, 2.191) < 0.001	1.009 (1.003, 1.015) 0.006	1.318 (1.108, 1.574) 0.002	1.663 (1.256, 2.216) < 0.001	1.214 (1.073, 1.376) 0.002	1.024 (1.010, 1.039) 0.001	1.024 (1.008, 1.041) 0.003
No	2925	1.642 (1.426, 1.895) < 0.001	1.010 (1.007, 1.013) < 0.001	1.411 (1.295, 1.538) < 0.001	1.886 (1.639, 2.175) < 0.001	1.235 (1.163, 1.312) < 0.001	1.027 (1.020, 1.034) < 0.001	1.031 (1.023, 1.039) < 0.001
*P* for interaction		0.513	0.805	0.762	0.689	0.969	0.982	0.507

adjusted for age, gender, COPD, HTN, DM, Smoking, HDL-C, LDL-C, HbA1c, SCr, Cystatin C, and SUA.

OR, Odds Ratio; CI, Confidence Interval; BMI, Body Mass Index; COPD, Chronic Obstructive Pulmonary Disease; HTN; Hypertension; DM, Diabetes Mellitus, HDL-C, High-Density Lipoprotein Cholesterol, LDL-C, Low-Density Lipoprotein Cholesterol; HbA1c, Glycated hemoglobin; SCr, Serum Creatinine; SUA, Serum Uric Acid.

### Mediating role of HbA1c

We assessed the mediating role of HbA1c in the relationship between the TyG index and its related indices (such as TyG-BMI, TyG-WC, TyG-WHtR, TyG-CI, TyG-WWI, TyG-BRI) and CAD using mediation modeling. After adjusting for variables including age, gender, Smoking, HTN, DM, COPD, HDL-C, LDL-C, SUA, HbA1c, SCr, and Cystatin C, HbA1c was found to be a significant mediator in the relationship between the TyG index and its combinations with obesity indicators and CAD.

The indirect effects of the TyG index and its related indices on CAD were analyzed, revealing that the mediating effect of each TyG indicator on CAD ranged from 13.20% to 27.86%. This suggests that different TyG indices contribute significantly to mediating the effect on CAD. **Table 7** presents the total effect, direct effect, indirect effect, and the proportion of the mediating effect.

**Table 7:** Mediation analysis of exploring the mediating effect of HbA1c.

Effects from	Total (95% CI) *P* value	Direct (95% CI) *P* value	Indirect (95% CI) *P* value
TyG to CAD	2.336e-03 (1.020e-03, 4.947e-03) < 0.001	1.685e-03 (7.823e-04, 3.393e-03) < 0.001	6.508e-04 (2.338e-04, 1.641e-03) < 0.001
HbA1c (Mediator)	Prop. Mediated (95% CI) *P* value	% of Total Effect	
	2.786e-01 (1.903e-01, 3.784e-01) < 0.001	27.86%	
	Total (95% CI) *P* value	Direct (95% CI) *P* value	Indirect (95% CI) *P* value
TyG-BMI to CAD	7.253e-04 (5.170e-04, 9.033e-04) < 0.001	5.638e-04 (4.193e-04, 6.733e-04) < 0.001	1.616e-04 (8.878e-05, 2.690e-04) < 0.001
HbA1c (Mediator)	Prop. Mediated (95% CI) *P* value	% of Total Effect	
	2.228e-01 (1.528e-01, 3.066e-01) < 0.001	22.28%	
	Total (95% CI) *P* value	Direct (95% CI) *P* value	Indirect (95% CI) *P* value
TyG-WC to CAD	1.971e-02 (1.380e-02, 2.523e-02) < 0.001	1.544e-02 (1.111e-02, 1.921e-02) < 0.001	4.266e-03 (2.376e-03, 6.766e-03) < 0.001
HbA1c (Mediator)	Prop. Mediated (95% CI) *P* value	% of Total Effect	
	2.165e-01 (1.534e-01, 2.925e-01) < 0.001	21.65%	
	Total (95% CI) *P* value	Direct (95% CI) *P* value	Indirect (95% CI) *P* value
TyG-WHtR to CAD	3.357e-02 (2.536e-02, 4.277e-02) < 0.001	2.720e-02 (2.085e-02, 3.368e-02) < 0.001	6.373e-03 (3.962e-03, 9.706e-03) < 0.001
HbA1c (Mediator)	Prop. Mediated (95% CI) *P* value	% of Total Effect	
	1.899e-01 (1.377e-01, 2.473e-01) < 0.001	18.99%	
	Total (95% CI) *P* value	Direct (95% CI) *P* value	Indirect (95% CI) *P* value
TyG-CI to CAD	1.466e-02 (1.054e-02, 1.856e-02) < 0.001	1.102e-02 (8.136e-03, 1.323e-02) < 0.001	3.646e-03 (2.143e-03, 5.859e-03) < 0.001
HbA1c (Mediator)	Prop. Mediated (95% CI) *P* value	% of Total Effect	
	2.486e-01 (1.818e-01, 3.366e-01) < 0.001	24.86%	
	Total (95% CI) *P* value	Direct (95% CI) *P* value	Indirect (95% CI) *P* value
TyG-WWI to CAD	1.455e-03 (1.024e-03, 1.933e-03) < 0.001	1.119e-03 (8.161e-04, 1.403e-03) < 0.001	3.355e-04 (1.856e-04, 5.617e-04) < 0.001
HbA1c (Mediator)	Prop. Mediated (95% CI) *P* value	% of Total Effect	
	2.306e-01 (1.654e-01, 3.096e-01) < 0.001	23.06%	
	Total (95% CI) *P* value	Direct (95% CI) *P* value	Indirect (95% CI) *P* value
TyG-BRI to CAD	5.826e-03 (5.059e-03, 6.347e-03) < 0.001	5.057e-03 (4.228e-03, 5.644e-03) < 0.001	7.688e-04 (5.311e-04, 1.047e-03) < 0.001
HbA1c (Mediator)	Prop. Mediated (95% CI) *P* value	% of Total Effect	
	1.320e-01 (9.012e-02, 1.869e-01) < 0.001	13.20%	

adjusted for age, gender, COPD, HTN, DM, Smoking, HDL-C, LDL-C, HbA1c, SCr, Cystatin C, and SUA.

CI, Confidence Interval; COPD, Chronic Obstructive Pulmonary Disease; HTN; Hypertension; DM, Diabetes Mellitus, HDL-C, High-Density Lipoprotein Cholesterol, LDL-C, Low-Density Lipoprotein Cholesterol; HbA1c, Glycated hemoglobin; SCr, Serum Creatinine; SUA, Serum Uric Acid.

## Discussion

This study, conducted on 3, 641 adult participants, identified significant associations between the TyG index and its related indices and the presence of CAD. Specifically, increases in TyG, TyG-BMI, TyG-WC, TyG-WHtR, TyG-CI, TyG-WWI, and TyG-BRI were positively correlated with an increased prevalence of CAD. The relationship between TyG and CAD was linear, while the relationships between TyG-BMI, TyG-WC, TyG-WHtR, TyG-CI, TyG-WWI, and TyG-BRI and CAD were non-linear, particularly at the following thresholds: TyG-BMI > 241.329; TyG-WC > 8.547; TyG-WHtR > 5.099; TyG-CI > 11.928; TyG-WWI > 97.285; TyG-BRI > 48.051. The predictive performance differences for TyG-WHtR (*P* = 0.019) and TyG-WWI (*P* = 0.036) were significant in both models, with the segmented regression model slightly outperforming the logistic regression model. Additionally, subgroup analyses across different strata consistently demonstrated significant associations between TyG index and its related indices and CAD risk. Finally, mediation analysis indicated that HbA1c played a significant mediating role in the relationship between the TyG index and its related indices and CAD, contributing up to 27.86%, further emphasizing the importance of HbA1c in predicting CAD risk.

As one of the major public health challenges worldwide, CAD continues to have high morbidity and mortality rates. The occurrence of CAD is closely related to multiple factors, particularly IR, a metabolic disorder that is strongly associated with high CAD risk. IR often interacts with other CVD risk factors such as HTN and hypercholesterolemia, forming a “dangerous combination” that complicates the prevention and treatment of CVD (19, 20). In recent years, the TyG index has been widely recognized in clinical practice as a reliable and specific marker of IR, and it has become a promising biomarker due to its significant association with CAD. For example, a prospective cohort study involving 23, 591 diagnosed CAD patients with a median follow-up period of 28 months found that for every 1-unit increase in the TyG index, the risk of all-cause mortality increased by 22% (HR = 1.22, 95% CI = 1.02, 1.45), and the risk of cardiovascular death increased by 61% (HR = 1.61, 95% CI = 1.17, 2.22) (21). Another community-based study confirmed that in the prediabetes stage (22), each 1-unit increase in the TyG index was associated with a 12% increased risk of cardiovascular events. Additionally, a real-world cohort study observed that in CAD patients undergoing lipid-lowering treatment, higher baseline TyG index and increases during the follow-up period were associated with poor lipid control, suggesting that the TyG index may reflect the sustained burden of IR on the vascular and metabolic systems (23). These findings are highly consistent with the significant positive correlation between the TyG index and CAD risk observed in this study. The mechanism underlying the association between TyG index and increased CVD risk remains unclear, but IR likely plays a critical role. IR leads to impaired glucose control and accelerates atherosclerosis through enhanced low-grade chronic inflammation and oxidative stress (5, 24, 25). Hyperglycemia-induced glycation reactions reduce the bioavailability of NO in the vascular endothelium, further impairing endothelial-dependent vasodilation and leading to endothelial dysfunction (26). Moreover, IR alters lipid metabolism, promoting cholesterol accumulation and fat deposition, which together contribute to the development of atherosclerosis (27, 28). Further research indicates that hyperinsulinemia, as a marker of IR, activates the sympathetic nervous system, increasing the secretion of adrenaline and noradrenaline, creating an excessive neurohumoral feedback loop that results in increased cardiac output and peripheral vascular resistance. This process exacerbates atherosclerosis and vascular stiffness, thereby increasing CVD risk (29, 30). As an alternative marker of IR, the TyG index not only provides early clues for CAD detection but also offers a potential biomarker for CAD risk stratification in clinical practice.

Furthermore, obesity can serve as a key indicator for assessing the risk of various obesity-related chronic diseases. Studies have clearly shown that central obesity contributes to the development of CVD through multiple mechanisms, including the secretion of various adipokines, the induction of chronic inflammatory responses, and endothelial dysfunction (31, 32). Indicators used to measure obesity include BMI, WC, WHtR, WWI, CI, and BRI. Obesity is often associated with HTN and abnormal lipid metabolism, significantly increasing the risk of CVD. Obesity may also independently contribute to the development of CVD and CVD-related mortality, particularly in relation to the distribution of body fat, regardless of other CVD risk factors (33). Subsequent studies have combined obesity-related indicators with the TyG index to derive new indices, aiming to explore whether these combinations could improve predictive performance. Among many studies, TyG and its combinations with obesity-related indicators, such as TyG-BMI, TyG-WC, and TyG-WHtR, can serve as surrogate markers for IR (34). In addition, Li et al. (35) showed that TyG-BMI is significantly associated with CVD incidence in the Chinese population (OR = 1.168, 95% CI = 1.040, 1.310). A study based on NHANES data from 1999 to 2018 (36) showed that TyG-WWI outperforms TyG, TyG-WC, and TyG-WHtR in predicting cardiovascular mortality, with an AUC of 0.694, compared to TyG’s AUC of 0.627. Dang et al. (14) reported that TyG-WHtR is the strongest predictor of cardiovascular mortality (HR = 1.66, 95% CI = 1.21, 2.29) and outperforms TyG and TyG-WC in diagnostic accuracy. A study involving 1, 145 Korean participants (37) showed that TyG-WC has better diagnostic performance than TyG and TyG-BMI in predicting coronary artery calcification progression.

Our study indicates that the TyG-related indices have a nonlinear association with CAD risk, with a more pronounced increase in risk above certain thresholds. One possible explanation is that once these thresholds are exceeded, the metabolic burden associated with IR may become more clinically significant, potentially through enhanced inflammation and oxidative stress. However, this mechanistic interpretation remains hypothetical and requires further experimental validation. This process promotes low-grade chronic inflammation and oxidative stress through alterations in lipid metabolism and fat distribution, further driving the formation of atherosclerosis. These metabolic disturbances, especially when combined with obesity-related indicators such as abdominal obesity, may exacerbate the atherosclerotic process and increase CAD risk. This finding also provides a threshold reference for clinical risk intervention. Additionally, this study examined HbA1c as a potential mediating factor. The mediation analysis suggested that HbA1c may partially explain the association between the TyG index and its combinations with obesity-related indicators and CAD risk, with the mediated proportion reaching up to 27.86%. However, given the cross-sectional nature of this analysis, these findings should be interpreted as statistical mediation rather than evidence of a causal pathway. Prospective studies are needed to confirm the temporal relationship. Furthermore, we assessed the performance of the TyG index and its related indices in predicting CAD risk using ROC curve analysis. There is still debate regarding which TyG-related indices optimally predicts cardiovascular outcomes. Our results show that, in terms of AUC values, the TyG index demonstrated slightly higher discriminative ability (AUC = 0.639) compared to most combination indices (AUC ranging from 0.578 to 0.623), although TyG-WHtR showed similar performance (AUC = 0.623). However, it is important to note that all AUC values were in the range of 0.58-0.64, indicating only modest discriminatory power. Therefore, none of these indices, including the TyG index alone, should be considered strong standalone predictors of CAD. One possible explanation for the observation that combining obesity indicators did not improve predictive performance is that the TyG index itself may already capture a substantial portion of the metabolic risk associated with CAD. Obesity indicators, while reflecting fat distribution, may not add independent predictive information beyond that provided by the TyG index in this specific population. Nevertheless, given the modest overall predictive performance, this finding should be interpreted cautiously. It does not necessarily imply that the TyG index is a “superior” predictor, but rather that simple multiplication with obesity indicators does not enhance its limited discriminative ability in this context. Additionally, although some combination indices demonstrated lower AUC values, their associations with CAD remained statistically significant in regression analyses, suggesting they may still capture relevant metabolic information. However, based on the current data, their clinical utility—particularly for risk prediction—appears limited. Future research should focus on validating these findings in prospective cohorts, exploring whether these indices offer incremental predictive value beyond established cardiovascular risk factors, and assessing their potential applicability in specific populations or clinical settings where certain anthropometric measurements are more readily available or clinically relevant. Such investigations will help clarify the role of these combined indices without overgeneralizing their use.

### Limitations

This study has several limitations. First, due to its retrospective observational design and cross-sectional analysis, the results can only reveal associations between the TyG index and its related indices with CAD risk, and cannot infer causality. Retrospective data lack a clear temporal sequence; therefore, the mediating role of HbA1c in this relationship should be interpreted with caution. The current findings only reflect statistical associations between variables rather than precise causal pathways. The mediation analysis is based on cross-sectional data, and the temporal sequence between exposure, mediator, and outcome cannot be determined; consequently, these results should be considered exploratory findings. Additionally, although we calculated the indirect-to-direct effect ratios for each TyG index (e.g., 0.386 for TyG), we did not report their 95% bootstrap confidence intervals, as calculating these intervals requires the joint distribution of the indirect and direct effects—information not directly provided by our current software. Given that the 95% bootstrap CIs for both the indirect and direct effects excluded zero, the statistical significance of these ratios can be indirectly inferred. Future prospective longitudinal studies are needed to further validate the causal nature of these associations, and studies employing software capable of reporting bootstrap CIs for the indirect-to-direct effect ratio are warranted to confirm our findings. Second, due to the retrospective design, over 10, 000 patients (more than half of the initial cohort) were excluded because of missing laboratory or anthropometric data. Such a large proportion of missing data represents a significant limitation, as it may introduce selection bias and affect the generalizability (external validity) of the study findings. We acknowledge that, due to data limitations, we were unable to perform comprehensive quantitative comparisons (e.g., sensitivity analyses) between the included and excluded populations to fully assess this bias. Excluded patients may differ systematically from the included population in key characteristics (e.g., more severe disease, more comorbidities, or poorer medication adherence), suggesting that our final analytic sample may represent a relatively “healthier” or better-managed subgroup. This situation could lead to underestimation of the true association between TyG-related indices and CAD. Third, although multiple covariates were adjusted for in the multivariable models, unmeasured confounding factors may still exist. First, due to the lack of systematically recorded medication information in the retrospective data, we were unable to adjust for the use of lipid-lowering agents (e.g., statins), antidiabetic medications, or antihypertensive drugs. These medications may simultaneously affect metabolic indices and CAD risk: for example, statins can lower triglyceride levels, thereby influencing TyG index calculation, while also reducing CAD risk; failure to adjust for such medication use could lead to overestimation or underestimation of the TyG-CAD association. Second, inflammatory markers (such as hs-CRP) were not included in routine testing, yet inflammatory response is an important mechanism linking IR to atherosclerosis. The presence of these unmeasured confounders may result in residual confounding, thereby affecting the accuracy of the results. Fourth, ROC analysis demonstrated that the TyG index and its related indices have limited discriminative ability, with AUC values ranging from 0.578 to 0.639, indicating only moderate predictive performance. Therefore, these indices alone may not provide strong predictive power for CAD and should be interpreted cautiously in clinical applications to avoid overstating their predictive value. Future research should explore their incremental value when combined with traditional cardiovascular risk factors and assess their actual contribution to risk stratification using metrics such as the net reclassification improvement. Fifth, the representativeness of the study population is limited. This study only included patients who underwent coronary angiography, and in clinical practice, patients are referred for this examination often because of high clinical suspicion of significant coronary stenosis. Additionally, all samples were from a single center in the Cangzhou area, which may limit the generalizability of the results. Therefore, our study population may not represent the general population or early asymptomatic patients, which could introduce selection bias. Validation studies in different regions, ethnicities, or populations will help assess the applicability of these indices in broader populations. In summary, although this study revealed significant associations between the TyG index and its related indices with CAD risk, the aforementioned limitations suggest that the findings should be interpreted with caution. Future well-designed prospective studies are needed to validate our findings and further clarify the specific clinical applications of these indices.

## Conclusion

This study identified significant associations between the TyG index and its related indices with the risk of CAD, with HbA1c playing a partial mediating role in this relationship. However, due to the inherent limitations of the retrospective design, these associations should not be inferred as causal. Furthermore, the predictive performance of the TyG-related indices was only moderate, and their clinical applicability requires further validation to avoid over-interpretation. Future prospective studies should evaluate the incremental value of these indices beyond traditional risk factors and explore their specific applications in cardiovascular risk stratification.

## Data Availability

The original contributions presented in the study are included in the article/supplementary material. Further inquiries can be directed to the corresponding author.
